# High-intensity interval training attenuates development of autoimmune encephalomyelitis solely by systemic immunomodulation

**DOI:** 10.1038/s41598-023-43534-8

**Published:** 2023-10-02

**Authors:** Yehuda Goldberg, Shir Segal, Liel Hamdi, Hanan Nabat, Nina Fainstein, Efrat Mediouni, Yarden Asis, Paschalis Theotokis, Ilias Salamotas, Nikolaos Grigoriadis, Abram Katz, Tamir Ben-Hur, Ofira Einstein

**Affiliations:** 1 Department of Physical Therapy, Faculty of Health Sciences, Ariel University, 40700 Ariel, Israel; 2 Department of Neurology, The Agnes Ginges Center for Human Neurogenetics, Hadassah – Hebrew University Medical Center, Jerusalem, Israel; 3 B’ Department of Neurology, AHEPA University Hospital of Thessaloniki, Thessaloniki, Greece; 4 Åstrand Laboratory, The Swedish School of Sport and Health Sciences, GIH, Stockholm, Sweden

**Keywords:** Immunology, Neuroscience, Medical research, Neurology

## Abstract

The impact of high-intensity interval training (HIIT) on the central nervous system (CNS) in autoimmune neuroinflammation is not known. The aim of this study was to determine the direct effects of HIIT on the CNS and development of experimental autoimmune encephalomyelitis (EAE). Healthy mice were subjected to HIIT by treadmill running and the proteolipid protein (PLP) transfer EAE model was utilized. To examine neuroprotection, PLP-reactive lymph-node cells (LNCs) were transferred to HIIT and sedentary (SED) mice. To examine immunomodulation, PLP-reactive LNCs from HIIT and SED donor mice were transferred to naïve recipients and analyzed in vitro. HIIT in recipient mice did not affect the development of EAE following exposure to PLP-reactive LNCs. HIIT mice exhibited enhanced migration of systemic autoimmune cells into the CNS and increased demyelination. In contrast, EAE severity in recipient mice injected with PLP-reactive LNCs from HIIT donor mice was significantly diminished. The latter positive effect was associated with decreased migration of autoimmune cells into the CNS and inhibition of very late antigen (VLA)-4 expression in LNCs. Thus, the beneficial effect of HIIT on EAE development is attributed solely to systemic immunomodulatory effects, likely because of systemic inhibition of autoreactive cell migration and reduced VLA-4 integrin expression.

## Introduction

Beneficial outcomes of exercise training (ET) in multiple sclerosis (MS) patients are well documented^1,2^. Various mechanisms have been suggested to underlie the beneficial effects of ET in experimental autoimmune encephalomyelitis (EAE), an animal model of MS^3^. Accordingly, in a series of studies, we utilized the passive transfer EAE model, that enabled us to differentiate between systemic immunomodulatory and direct neuroprotective effects of ET, to examine the impact of various training paradigms on the progression of EAE^4–8^.

We found that high intensity continuous training (HICT) was superior to moderate intensity continuous training in yielding favorable effects on disease progression^4,5,7,8^. We demonstrated that both direct effects of HICT on the CNS and indirect effects via systemic immunomodulation mitigate disease progression^4,5,7,8^. Specifically, we found that HICT inhibits several key molecules involved in encephalitogenic lymph node cell (LNC) migration into the CNS^7^, and modulates the properties of the blood–brain-barrier (BBB) to maintain the integrity of the BBB following EAE induction^7^. Moreover, the results indicated that ~ 70% of the beneficial effects of HICT on disease development derive from systemic immunomodulation and ~ 30% from direct protection of the CNS^7^.

HIIT has become a popular and efficient mode of ET^9^. Its popularity derives from studies that show similar or superior physiological and metabolic adaptations achieved by HIIT in a shorter time duration than HICT^10–12^. The beneficial effects of HIIT have also been documented in autoimmune diseases such as rheumatoid arthritis, as well as diabetes^13–15^. Several studies also indicate that HIIT improves fitness in people with MS^16^. However, the direct effects of HIIT on the CNS in autoimmune neuroinflammation have not been studied. Considering that HICT results in direct neuroprotective effects and attenuates development of EAE^7^ and that HIIT enhances exercise performance more than HICT^6^, we suspected that HIIT can also impact neuroprotection, possibly even more positively than HICT.

Therefore, in this study we investigated the direct effects of HIIT on the CNS, as well as the development of EAE. Additionally, we examined potential mechanisms whereby HIIT exerts its effects on autoimmune neuroinflammation.

## Materials and methods

### Experimental animals

Female SJL/JCrHsd mice (6–7 weeks of age) were purchased from Envigo Inc, Israel. Animal experimentation was approved by the Institutional Animal Care and Use Committee. The studies were conducted in accordance with the United States Public Health Service's Policy on Humane Care and Use of Laboratory Animals.

### Experimental design

The PLP_139–151_ transfer EAE model and the HIIT treadmill running program were utilized as previously described^6^.

**Figure 1:**
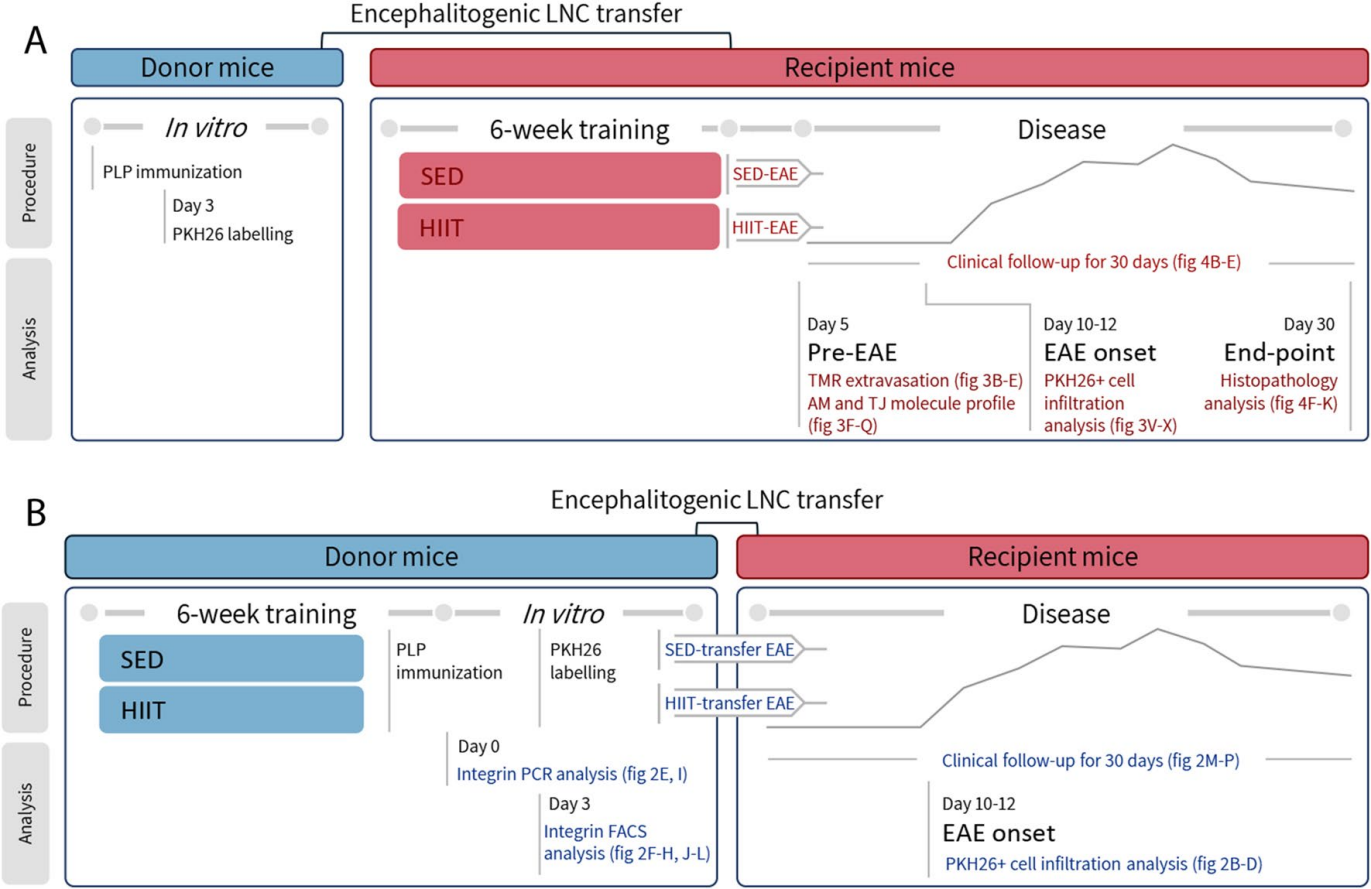
Experimental design to study the direct neuroprotective and systemic immunomodulatory effects of HIIT on EAE. The transfer EAE model was utilized to differentiate between the effects of HIIT directly on the central nervous system (CNS) (**A**) and on systemic autoimmunity (**B**). (**A**) Healthy mice were subjected to a HIIT treadmill running program or sedentary (SED) period and served as recipients to further develop EAE. Another group of donors was immunized with proteolipid protein (PLP), and their lymph node cells (LNCs) were further stimulated for 72 h in culture with PLP. Encephalitogenic LNCs were labelled with the fluorescent marker PKH26 and injected into trained (HIIT-EAE) or SED-control (SED-EAE) recipient mice. HIIT-EAE and SED-EAE mice were scored daily for neurological symptoms, and histopathology analysis of their spinal cords was performed at the end of the clinical follow-up. In some experiments, at EAE onset, the spinal cords of SED-EAE and HIIT-EAE mice were examined for PKH26+ cell infiltration. Additionally, at day 5 post LNC transfer (pre-EAE), the spinal cords of HIIT-EAE and SED-EAE were analyzed for blood–brain-barrier (BBB) permeability [by 5-(and 6)-tetramethylrhodamine biocytin (TMR) extravasation] and for adhesion molecules (AM) and tight junction (TJ) profile. (**B**) Healthy donor mice were subjected to a HIIT treadmill running program. SED mice served as controls. HIIT and SED mice were immunized with PLP peptide, their LNCs were stimulated for 72 h in culture with PLP and injected into naïve recipients that developed EAE (HIIT-transfer-EAE and SED-transfer EAE, respectively). HIIT-transfer-EAE and SED-transfer-EAE mice were scored daily for neurological symptoms. In some experiments, prior to their transfer, encephalitogenic LNCs were labelled with PKH26 for cell infiltration analysis in spinal cords of recipient mice at EAE onset. Additionally, encephalitogenic LNCs from HIIT and SED mice were analyzed for their integrin properties at day of isolation [day 0, RT-PCR analysis] and following in vitro stimulation with PLP [day 3, fluorescent- activated cells sorting (FACS) analysis].

The direct neuroprotective effects of HIIT were assessed in HIIT and SED recipient mice following autoimmune LNC transfer (Fig. 1A). Healthy mice were subjected to a defined HIIT treadmill running program, followed by injection of PLP-reactive, encephalitogenic LNCs from donor mice. SED mice were injected with the same PLP-reactive encephalitogenic cells and served as controls. The direct effect of the HIIT program on the central nervous system (CNS) of the recipient mice was examined by: (1) the severity of EAE and CNS pathology ; (2) the permeability of the BBB to 5-(and 6)-tetramethylrhodamine biocytin (biocytin TMR) and the expression of adhesion and the tight junction molecules at the pre- EAE stage; and (3) and the permeability of the BBB to PKH26- labelled encephalitogenic cells at EAE onset.

The modulatory effects of HIIT on systemic autoimmunity and on the migratory potential and encephalitogenicity of proteolipid protein (PLP)- reactive LNCs were examined in vivo following HIIT and sedentary (SED) donor mice- derived autoimmune cell transfer in recipient groups and in vitro (Fig. 1B). Healthy mice were subjected to a 6-wk defined HIIT treadmill running program (see below). Another group of SED mice served as controls. HIIT and SED mice were immunized with a PLP peptide, their inguinal lymph nodes were removed and LNCs were cultured in vitro for 72 h with PLP. PLP- reactive LNCs from HIIT and SED mice were labeled with PKH-26 red fluorescent cell linker (Fig. 1B, left panel) and injected into recipient mice (Fig. 1B, right panel). The effects of the HIIT program on the systemic autoimmune process in the donor mice was examined by: (1) examination of the clinical severity of EAE in recipient mice, following transfer of encephalitogenic LNCs from HIIT- versus SED donor mice (Fig. 1B, right panel); (2) counts of injected PKH26- labeled encephalitogenic cells from HIIT- versus SED donor mice in the CNS of recipient mice at EAE onset (Fig. 1B, right panel); and (3) integrin gene and surface expression analyses of PLP- reactive LNCs at the day of excision and following 72 h in culture (Fig. 1B, left panel).

### High-intensity interval training (HIIT) treadmill running program

Healthy mice underwent 6-weeks of HIIT treadmill running, including pre- and post- training performance tests on a 5-lane treadmill designed for mice (Panlab Harvard Apparatus, USA) as previously described^6^.

### Passive transfer experimental autoimmune encephalomyelitis (EAE)

**Figure 2:**
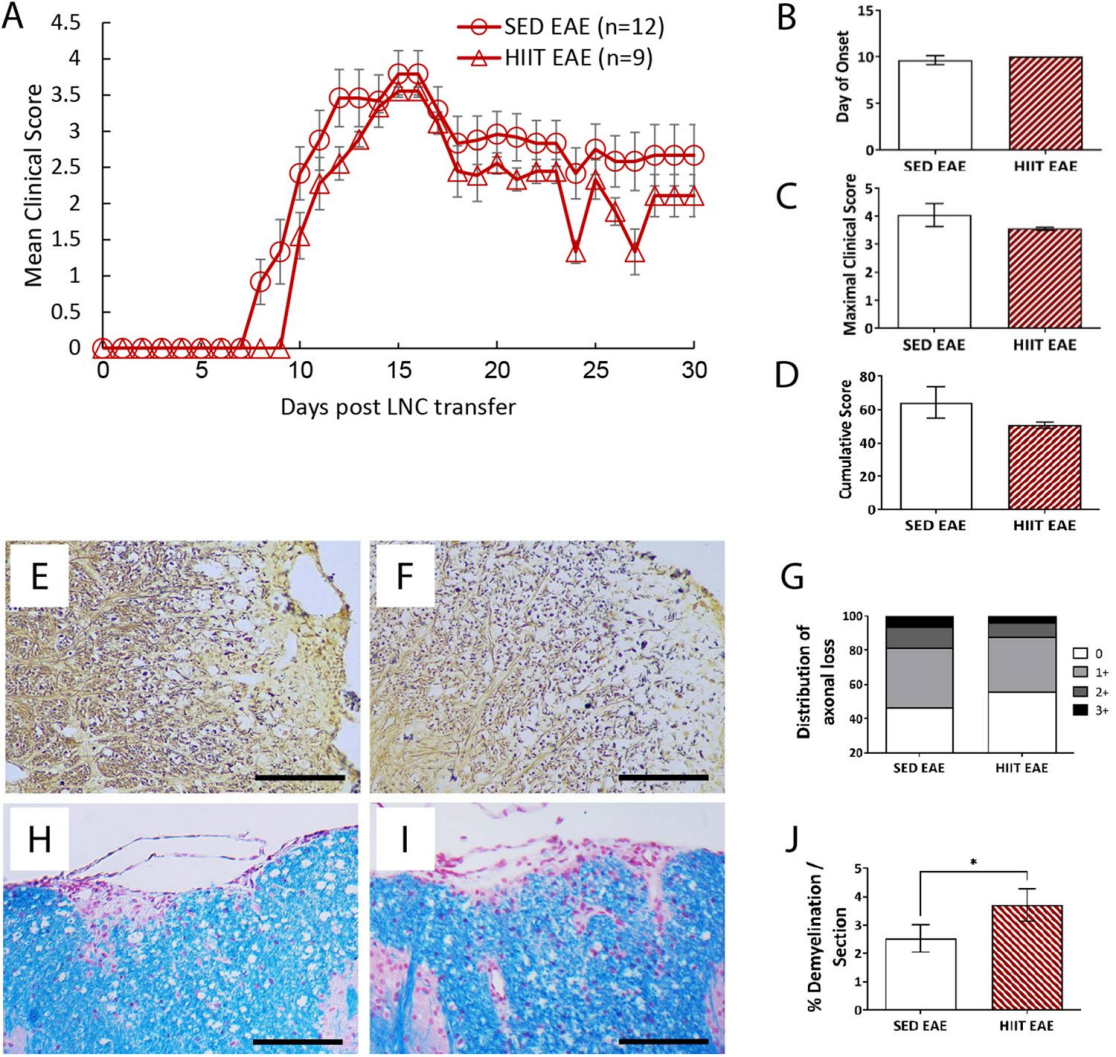
HIIT does not induce direct neuroprotection in recipient mice in a transfer model of EAE. The direct effects of HIIT on the clinical course and pathology of the central nervous system (CNS) in transfer EAE model was investigated (see Fig. 1A). Clinical course (**A**) and clinical parameters (**B**–**D**) of transfer EAE in HIIT-EAE and SED-EAE mice along 30 days of clinical follow-up. The severity of EAE was scored on 0–6 scale. (**E**–**J**) Histopathology analyses at the end of the clinical follow up for axonal loss (**E**–**G**) and demyelination (**H**–**J**) on cross sections of the spinal cords of SED-EAE (**E**, **H**) and HIIT-EAE (**F**, **I**) mice. Transfer of encephalitogenic LNCs to HIIT recipients did not affect the clinical severity of EAE (**A**–**D**), nor the degree of axonal pathology (**E**–**G**) compared to SED-EAE mice. The degree of demyelination was significantly increased in HIIT-EAE (**J**) mice compared to SED-EAE group (**I**, **K**). Scale bars = 100 μm; Data are mean ± SE. *p < 0.05.

**Figure 3:**
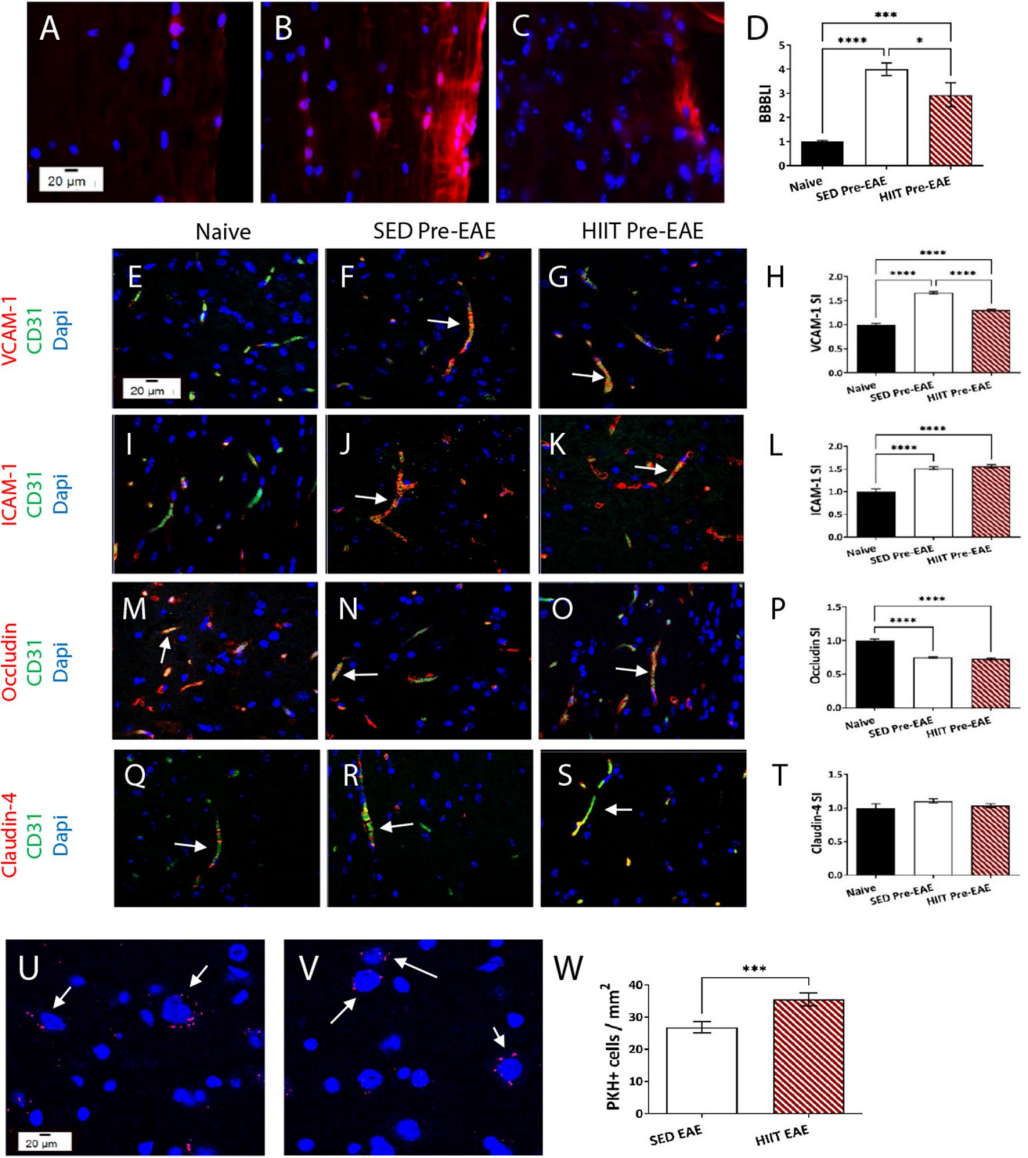
HIIT partly modulates the BBB permeability in recipient mice in a transfer model of EAE. The direct effects of HIIT on the blood–brain-barrier (BBB), and autoimmune cell infiltration into the central nervous system (CNS) in transfer EAE model were examined (see Fig. 1A). Biocytin-TMR fluorescence in longitudinal spinal cord sections of naïve (**A**), SED Pre-EAE (**B**) and HIIT Pre-EAE (**C**) mice. Additionally, CD31+ endothelial cells in spinal cords of HIIT Pre-EAE and SED Pre- EAE recipients were analyzed for the expression of vascular cell adhesion molecule (VCAM)-1 (**E**–**H**) and intercellular adhesion molecule (ICAM)-1 (**I**-**L**), and tight junction molecules occludin (**M**–**P**) and claudin-4 (**Q**–**T**) expression. In some experiments, prior to their injection to HIIT and SED recipients, encephalitogenic LNCs from donor mice were labelled with the fluorescent marker PKH26 (**U**–**W**; HIIT EAE and SED EAE, respectively). At day of EAE onset, longitudinal spinal cord sections of HIIT-EAE (**V**) and SED-EAE (**U**) were analyzed for PKH26 + cell infiltration. The number of PKH26+ infiltrating encephalitogenic cells in HIIT-EAE mice were significantly higher than in SED-EAE mice (**W**). Biocytin-TMR—red; Occludin, claudin-4, VCAM-1, ICAM-1—red; CD31—green; PKH26—red; Dapi—blue. (**E**): BBB leakage index (BBBLI) = relative to naïve controls. (**D**,**H**,**L**,**P**,**T**): Stimulation Index (SI) = relative to naïve controls. Data are mean ± SE. *p < 0.05, ***p < 0.001, ****p < 0.0001.

**Figure 4:**
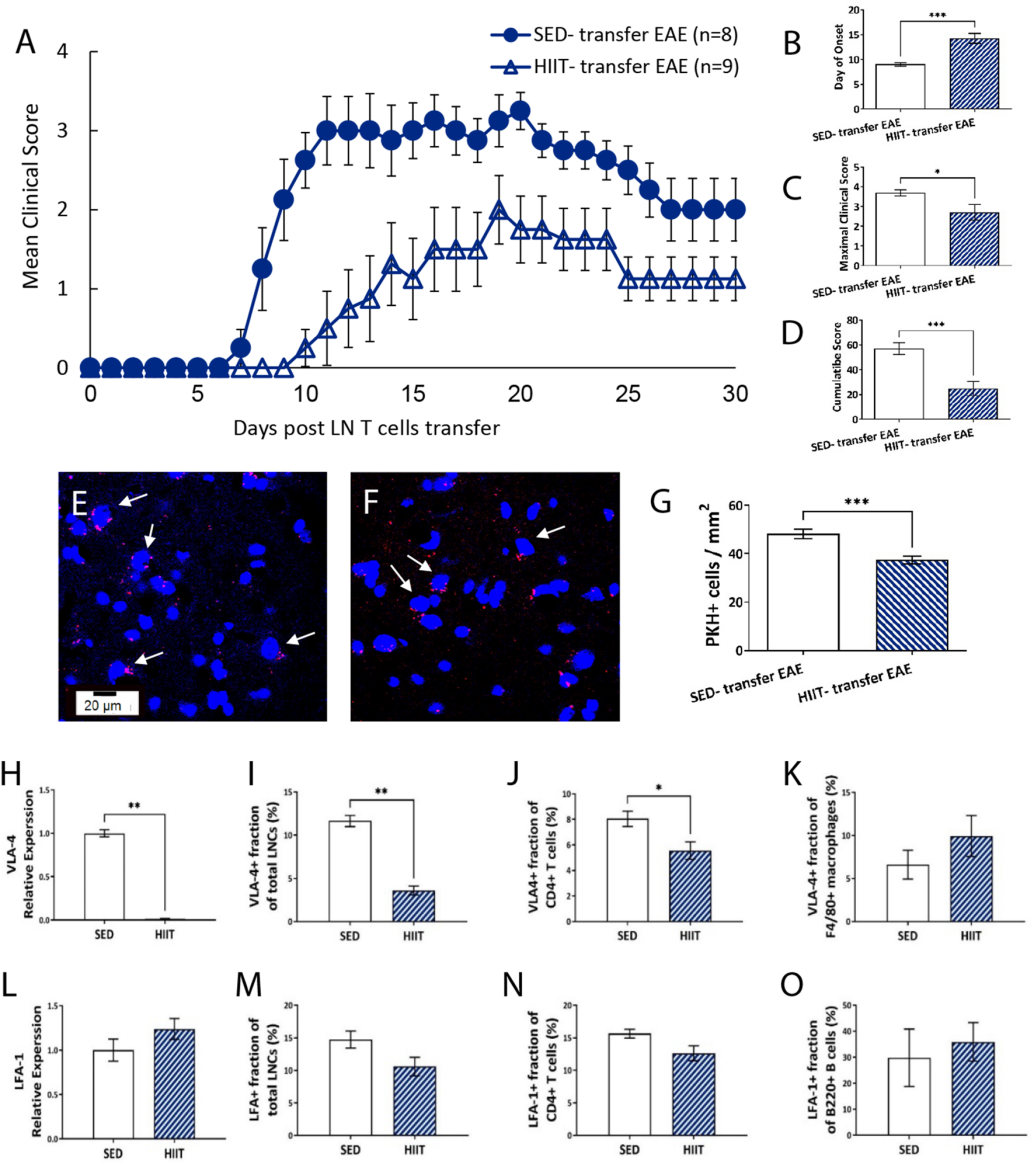
HIIT induces systemic immunomodulation and inhibits encephalitogenic LNC migration in a transfer model of EAE. (**A**) The systemic immunomodulatory effects of HIIT on encephalitogenic lymph node cells (LNCs) was investigated (see Fig. 1B). Clinical course (**A**) and clinical parameters (**B**–**D**) in SED- transfer EAE and HIIT- transfer EAE mice. The severity of EAE in recipient mice was scored on a 0–6 scale. Transfer of encephalitogenic LNCs derived from HIIT PLP-immunized mice to naïve recipients induced a significantly milder EAE course (**A**–**D**). At day of EAE onset, longitudinal spinal cord sections of SED- transfer EAE (**E**) and HIIT- transfer EAE (**F**) recipient mice were analyzed for PKH26+ cell infiltration. The number of PKH+ infiltrating encephalitogenic cells in HIIT-transfer EAE mice was significantly lower than in SED-transfer EAE mice (**G**). Encephailtogenic LNCs from HIIT and SED mice were analyzed in vitro for integrin expression (**H**–**O**). Real time- PCR analysis of freshly isolated LNCs for mRNA levels of very late antigen (VLA)-4 (xH) and lymphocyte function associated antigen (LFA)-1 (L). Flow cytometry analysis after 72 h of in vitro stimulation with PLP peptide of VLA-4 (**I**–**K**) and LFA-1 (**M**–**O**) expression in total LNCs (**I**,**M**), CD4+ T cells (**J**, **N**), F4/80+ macrophages (**K**) and B220 B cells (**O**). HIIT in PLP- immunized mice induced a marked reduction in mRNA levels of VLA-4 in LNCs (**H**) and in the fraction of VLA-4+ LNCs (**I**) and CD4+ T cells (**J**). PKH26—Red, Dapi—Blue. Data are mean ± SE. (**E**,**I**): Relative expression to SED group = 1. *p < 0.05, **p < 0.01, ***p < 0.001.

The PLP_139–151_ transfer EAE model was utilized as previously described^4–8^. EAE was induced either in recipient HIIT (HIIT-EAE, HIIT Pre-EAE) and SED (SED-EAE, SED Pre EAE) mice by transfer of encephalitogenic cells obtained from PLP-immunized donor mice (Figs. 1A, 2 and 3), or in naïve recipient mice by transfer of encephalitogenic cells from PLP-immunized HIIT (HIIT-transfer-EAE) and SED (SED-transfer-EAE) mice (30 × 10^6^ cells per mouse, Figs. 1B and 4).

### Clinical evaluation of EAE

HIIT-EAE (n = 9), SED-EAE (n = 12), HIIT-transfer-EAE (n = 10) and SED-transfer-EAE (n = 10) recipient mice developed EAE and were scored daily for neurological symptoms up to 30 days after EAE induction as follows: 0—asymptomatic; 1—partial loss of tail tonicity; 2—atonic tail; 3 -hind leg weakness, difficulty to roll over, or both; 4—hind leg paralysis; 5—four leg paralysis; 6—death due to EAE. To assess disease severity, day of onset, maximal clinical score, and cumulative score (“area under curve”) were calculated for each mouse at the end of the follow-up. The cumulative score was calculated in individual mice by summation of the daily clinical scores over the entire follow-up period.

### Histopathology analyses of the CNS in EAE

At the end of the clinical follow-up, a group of SED-EAE and HIIT-EAE mice (Figs. 1A and 2E–J, n  =  6/group) were sacrificed for histopathology analyses as previously described^4–8,17^. Mice were anesthetized with a lethal dose of sodium pentobarbital and subjected to perfusion via the ascending aorta with ice cold phosphate-buffered saline followed by 4% paraformaldehyde. The tissues were dissected and post-fixated in 4% paraformaldehyde for 24 h. Serial 6 μm paraffin embedded transverse sections were obtained from mid-cervical, mid-thoracic and mid-lumbar levels of the spinal cord. Sections underwent routine histology staining. Myelin maintenance was assessed using Luxol fast blue (LFB/Solvent Blue 38; Sigma, S3382-25G) counterstained with nuclear fast red (Sigma; N8002). Axon maintenance was evaluated using Bielschowsky silver impregnation using silver nitrate (Chem-lab CL00.2614.0250) and ammonia solution (32%, Merck; 1.05426.1000). For each stain, the whole white matter of three sections per mouse was quantified in a blind fashion, one section per spinal cord level. Demyelination was assessed by calculating the area of LFB loss. For chronic axonal damage, the area of reduced axonal density in Bielschowsky silver staining was assessed and scored as follows: 0 = normal/even silver stain throughout the white matter; 1 = small sporadic areas in the white matter that lack silver stain; 2 = small but frequent areas in the white matter that lack silver stain; 3 = extensive loss of silver stain throughout the white matter. All pathology measurements were performed by using the Image J software analysis (ver. 1.51H, NIH, USA).

### Assessment of autoimmune cell penetration into the CNS

Lymph nodes were excised from naïve (Fig. 1A) or HIIT and SED (Fig. 1B) donor mice at 10 days after PLP immunization. LNCs were cultured for 72 h as single cell suspensions with 10 μg/ml PLP peptide. PLP- reactive encephalitogenic cells from the donor mice were labelled by PKH26 red fluorescent cell linker kit, according to manufacturer ‘s instructions (SIGMA-ALDRICH). PKH26- labeled cells were injected intraperitoneally to HIIT-EAE and SED-EAE recipient mice (Fig. 1A and 3U–W) or HIIT-transfer-EAE and SED-transfer-EAE (Figs. 1B and 4E-G; 30 × 10^6^ cells per mouse, n = 5/group). At the day of clinical onset, recipient mice were sacrificed by intracardial perfusion with 4% paraformaldehyde. Thereafter, spinal cords were removed and serial 10 μm longitudinal frozen sections were prepared, and counterstained with DAPI. For each mouse, a total of 100 microscopic images (X40 magnification) from six sections at 60 μm apart were captured and quantified in a blind fashion. PKH+ cells were counted in the spinal cord parenchyma and reported as total average number ± SEM per square millimeter. Measurements were performed by using the Image J software analysis (ver. 1.51H, NIH, USA).

### Analyses of blood–brain-barrier properties and permeability

The BBB properties and permeability were evaluated in spinal cords of HIIT and SED recipient mice after transfer of encephalitogenic PLP-reactive cells from donor mice (Fig. 1A) by: (1) 5-(and 6)-tetramethylrhodamine biocytin (biocytin-TMR) extravasation assessment (HIIT pre-EAE, n = 6; SED pre- EAE, n = 5; naïve controls, n = 3; Fig. 3A–D); (2) adhesion and tight junction molecule expression (HIIT pre-EAE, n = 5; SED pre- EAE, n = 5; naïve controls, n = 3; Fig. 3E–T); and (3) PKH26- labelled encephalitogenic cell penetration (HIIT-EAE, SED-EAE, n = 5/group; Fig. 3U–W) .

### Assessment of biocytin-TMR leakage

The integrity of the BBB was examined by the biocytin- TMR (Invitrogen, Thermo Fisher Scientific) extravasation method as previously described^7^ (Figs. 1A and 3A–D). At 5 days following transfer of encephalitogenic cells, prior to appearance of clinical symptoms (pre- EAE), a total of 1 mg of biocytin-TMR diluted in 100 μL PBS per mouse was injected into the tail vein of HIIT Pre-EAE (n = 6), SED Pre- EAE (n = 5), and naïve controls (n = 3). Thirty minutes after injection, animals were sacrificed by intracardial perfusion with 4% paraformaldehyde, spinal cords were extracted and serial 10 μm longitudinal frozen sections were prepared. Nuclear counterstain was performed using DAPI (Vector Laboratories). Biocytin-TMR extravasation was quantified by measuring the mean fluorescence intensity (MFI) in spinal cord sections. Images of six sections from each spinal cord, 60 μm apart, were captured uniformly using identical laser intensity, exposure times and magnification, and thresholded according to spinal cord from biocytin-TMR- injected (positive control) and non-injected (negative control) naïve mice. Areas that exceeded the threshold levels were defined as leakage area. Group average MFI ± SEM was calculated and reported as ratios compared to MFI of naïve mice sections (blood–brain-barrier leakage index—BBLI). Measurements were performed by using the Image J software analysis (ver. 1.51H, NIH, USA).

### Assessment of adhesion and tight junction molecule expressions

Adhesion and tight junction molecule expression assessments were performed as previously described^7^ (Figs. 1A and 3E–T). Immunofluorescence was performed on spinal cords of HIIT Pre-EAE, SED Pre- EAE mice (n = 5/group) and naïve controls (n = 3) for VCAM-1 (monoclonal anti-mouse VCAM-1, sc-13160, 1:800, Santa cruz), ICAM-1 (monoclonal anti-mouse ICAM-1, sc-107, 1:200, Santa cruz), occludin (monoclonal anti-mouse occludin, sc-133256, 1:600, Santa cruz) and claudin-4 (monoclonal anti-mouse claudin-4, sc-376643, 1:800, Santa cruz). To identify blood vessels, sections were double stained with CD31 (rat anti-mouse CD31, 550274, 1:800, BD-Pharmingen) and nuclear counterstain was performed using DAPI. Ten microscopic field images (X40 magnification) of three sections in each spinal cord at 60 μm apart were captured as described above. Stained areas around blood vessels in each image were marked. Group average MFI ± SEM was calculated and reported as ratio to stained area in spinal cords of naïve mice. Measurements were performed by using the Image J software analysis (ver. 1.51H, NIH, USA).

### In vitro analyses of encephalitogenic LNCs

Lymph nodes were excised from HIIT or SED mice at 10 days after PLP immunization and cultured for 72 h as single cell suspensions with 10 μg/mL PLP peptide (Fig. 1B). LNCs were analyzed at day of excision for integrin gene determination using real time- polymerase chain reaction (RT-PCR, Fig. 4H,L), and following 72 h in culture for surface integrin expression using flow cytometry (Figs. 4I–K,M–O), as previously described^7^.

### Gene determination of integrins

Total RNA was prepared using the RNeasy Plus Mini Kit (QIAGEN) from freshly isolated LNCs that were excised from HIIT and SED mice 10 days after PLP immunization (Fig. 1B and 4H, L; n = 5/group). cDNA was prepared from 300 ng total RNA using qScript cDNA Synthesis Kit (Quanta Biosciences), according to manufacturer’s instructions. Semiquantitative real-time PCR (RT-PCR) was performed using the PerfeCTa SYBR Green FastMix, ROX (Quanta Biosciences). For real-time (RT) PCR, the reaction mixture included 1 μL of cDNA, 300 nmol/L concentrations of the appropriate forward and reverse primers (Syntezza, Israel), and 5 μL of SYBR green mix PerfeCTa SYBR Green FastMix Rox (Quanta Biosciences) in a total volume of 10 μL. Gene amplification was carried out using the GeneAmp 7000 PCR system (Applied Biosystems). The gene expression results were normalized to the TBA gene. Two independent experiments were repeated in triplicate and are presented as the SE of mean. For the mRNA quantification, the fold change was normalized to the TBA transcript (ΔCT).

### Flow cytometry for surface expression of integrins

LNCs from PLP- immunized HIIT and SED mice were analyzed for surface integrin expression following their activation in vitro for 72 h in culture with PLP peptide, using flow cytometry (Figs. 1B and 4I–K,M–O; n = 8/group). The following antibodies were used: FITC-labeled anti-CD4 (anti- mouse CD4, 100,405, BioLegend) and PE-labeled anti- VLA-4 (anti- mouse CD49d, 103,607, Biolegend), APC-labeled anti- LFA-1 (anti- human CD11a/CD18, 363,409, BioLegend), FITC-labeled anti-F4/80 (anti- mouse F4/80, 123,107, BioLegend), PE-labeled anti-B220 (anti- CD45R, 45-0452-82, eBiosciense). The fraction of VLA-4+ or LFA-1+ cells out of total LNCs was calculated. Double staining with anti-VLA-4 and anti-CD4 or anti-F4/80 was used to identify the fraction of VLA-4+ T cells or macrophages, respectively. Double staining with anti-LFA-1 and anti-CD4 or anti-B220 was used to identify the fraction of LFA-1+ T cells or B cells, respectively. In all flow cytometry experiments, cells were pre-coated with anti–mouse CD16/CD32 (BD Pharmingen), as an Fc blocker, to block non-specific binding. In early experiments antibodies were tested for their specificity with an isotype control. All samples were analyzed in a Cytomics FC 500 apparatus (Beckman Coulter, Life Science) using the CXP analysis software (ver. 2.1; Informer Technologies, Inc).

### Statistical analyses

Normality of distribution of variables was tested by the Shapiro–Wilk test followed by the appropriate statistical test for comparison. Two experimental groups were compared using the unpaired Student’s *t* test or the two-tailed Mann–Whitney test, according to the normality test. When comparing > 2 mean values, a one-way analysis of variance (ANOVA) was used. If a significant F ratio was found, then the Newman-Keuls multiple comparison test was used to identify the location of significance. Data were analyzed in GraphPad Prism software v.5. Differences were considered statistically significant at *p* < 0.05. All data are presented as mean ± standard error of mean (SEM).

### Ethics approval

Animal experimentation was approved by the institutional ethics committee, approval number IL-185-11-19. The study is reported in accordance with ARRIVE guidelines.

## Results

### HIIT does not affect the clinical severity and tissue pathology in recipient mice

First, we evaluated if HIIT affected the clinical severity and pathological process of EAE (Fig. 2). Transfer of encephalitogenic LNCs derived from PLP-immunized donor mice induced a similar clinical course of EAE in HIIT-EAE and SED-EAE recipient mice (Fig. 2A). HIIT prior to encephalitogenic LNC transfer did not affect the day of onset (Fig. 2B), the maximal clinical score (Fig. 2C), nor the cumulative score (Fig. 2D). Accordingly, EAE induction did not impact the degree of axonal pathology in spinal cords of HIIT-EAE mice, compared to SED-EAE mice (Fig. 2E–G). However, HIIT induced an ~ 40% increase in demyelinated areas (Fig. 2H–J).

### HIIT induces partial reduction in BBB permeability

Since HIIT did not affect the clinical severity in EAE recipient mice, we studied the direct effects of HIIT on the BBB. To that end, encephalitogenic LNCs were transferred to HIIT and SED recipient mice (Fig. 1A). Since systemically administered PLP-reactive encephalitogenic LNCs encountered the CNS that had already been modulated by HIIT, we first examined the integrity of the BBB at day 5 post- encephalitogenic LNC transfer, a time point of initial invasion of autoimmune cells into the CNS, and prior to clinical onset (HIIT pre-EAE vs. SED pre-EAE), by biocytin-TMR extravasation (Figs. 1A and 3A–D) and tight junction and adhesion molecule expression (Figs. 1A and 3E–T). Transfer of PLP- reactive encephalitogenic LNCs to sedentary mice induced a four-fold increase in extravasation of biocytin-TMR in spinal cords of SED pre-EAE mice (Fig. 3B) versus naïve healthy mice (Fig. 3A,D). HIIT induced an ~ 30% reduction of the increase in extravasation of biocytin-TMR (Fig. 3D,E).

To examine the direct effect of HIIT on the profile of endothelial cells of the blood vessels, CD31+ endothelial cells in spinal cords of naïve healthy mice, SED- and HIIT- pre EAE mice were double stained with VCAM-1 (Fig. 3E–G), ICAM-1 (Fig. 3I–K) occludin (Fig. 3M–O) and claudin-4 (Fig. 3Q–S). Immunofluorescent analysis indicated that the basal expression of VCAM-1 and ICAM-1 in naïve mice (Fig. 3E,I) was markedly increased following encephalitogenic LNC transfer in SED pre- EAE mice (Fig. 3F,H,J,L). The increase of VACM-1 was significantly decreased in HIIT pre-EAE mice (Fig. 3G,H), whereas no significant difference was observed in the expression of ICAM-1 between the HIIT- and SED pre-EAE groups (Fig. 3J–L). Occludin expression in CD31 + endothelial cells was reduced by ~ 20% following transfer of encephalitogenic LNCs in both SED pre- EAE mice (Fig. 3N) and HIIT pre- EAE mice (Fig. 3O), compared to naïve healthy mice (Fig. 3M,P). Transfer of encephalitogenic LNCs had no effect on claudin-4 expression in SED pre- EAE (Fig. 3R) and HIIT pre- EAE (Fig. 3S) mice versus naïve controls (Fig. 3Q,T).

Thus, HIIT induced a reduction in BBB permeability to biocytin-TMR and prevented the induction of VCAM-1 expression in CD31+ endothelial cells, but did not affect ICAM-1, occludin and claudin-4 expressions in CD31+ endothelial cells.

### HIIT does not induce reduction in infiltration of autoreactive cells into the CNS

Further, the direct effects of HIIT on the permeability of the BBB to autoreactive LNCs was examined by counts of injected PKH + encephalitogenic LNCs derived from donor mice at day of EAE onset in recipient mice (Fig. 3A,U–W; HIIT EAE vs. SED EAE). Surprisingly, the number of PKH + infiltrating encephalitogenic LNCs in HIIT EAE mice (Fig. 3V) was ~ 25% higher than in SED EAE mice (Fig. 3U,W).

Thus, despite the HIIT-mediated reductions in biocytin-TMR extravasation and VCAM-1 expression, encephalitogenic LNC infiltration into the CNS was increased rather than decreased.

### HIIT induces systemic immunomodulation in donor mice to attenuate the clinical severity in recipient EAE mice

Owing to the unexpected negative effects of HIIT on the CNS, as well as a lack of a positive effect on development of EAE, we examined the effects of HIIT on systemic immunomodulation. First, we confirmed that HIIT has a positive effect on EAE development in recipient mice (Fig. 4A–D; HIIT- transfer EAE vs. SED- transfer EAE). Indeed, transfer of encephalitogenic LNCs from PLP-immunized HIIT donor mice induced a milder clinical course of EAE in recipient mice, compared to mice that received encephalitogenic LNCs from PLP-immunized SED donor mice (Fig. 4A). The average onset of disease in the HIIT- transfer EAE group was delayed by > 5 days (Fig. 4B), and the average maximal clinical score and the cumulative score were significantly decreased by ~ 30% (Fig. 4C) and ~ 60% (Fig. 4D), respectively, compared to SED- transfer EAE mice. To assess the mechanisms whereby HIIT exerted its positive effects on EAE development, we examined autoimmune cell migration and expression of integrins.

### HIIT induces inhibition of the migratory capacity of encephalitogenic LNCs

The immunomodulatory effects of HIIT on the migratory capacity of encephalitogenic LNCs into the CNS were examined (Figs. 1B and 4E–G). HIIT and SED- derived PLP- reactive LNCs were labeled with PKH26, injected into healthy recipients, and counted in spinal cord sections at day of EAE onset (Fig. 1B). The number of PKH+ infiltrating encephalitogenic LNCs derived from PLP-immunized HIIT donor mice in spinal cords of recipient EAE mice (HIIT- transfer EAE) was > 20% lower than in recipients that were injected with PLP- reactive LNCs derived from SED mice (SED- transfer EAE; Fig. 4E–G).

### HIIT induces reductions in VLA-4 integrin expression in PLP-reactive encephalitogenic LNCs

Since recipient EAE mice that were transferred with PKH + HIIT- derived encephalitogenic LNCS exhibited reduction in the number of donors’ infiltrating cells in vivo, we investigated whether this was associated with reduced integrin expression on PLP-reactive LNCs in vitro (Figs. 1B and 4H–O). First, integrin gene expression of freshly isolated LNCs was analyzed (Fig. 4H,L). HIIT essentially abolished the increases in VLA-4 mRNA levels versus SED controls (Fig. 4H), whereas mRNA levels of LFA-1 integrin were not affected (Fig. 4L). Then, integrin surface expression in LNCs following re-stimulation with PLP was examined (Fig. 4I–K,M–O). The fractions of VLA-4+ LNCs (Fig. 4I) and VLA-4+ , CD4+ T cells (Fig. 4J) were significantly reduced by > 60% and ~ 40%, respectively, in HIIT PLP-immunized mice. However, the fraction of VLA-4+/F4/80+ macrophages (Fig. 4K) and the fractions of LFA-1+ cells (Fig. 4N,O) were not affected by HIIT. Thus, HIIT reduces the capacity of encephalitogenic LNCs to migrate into the CNS, with a marked reduction in the expression of VLA-4 integrin.

## Discussion

This study investigated the direct effects of HIIT on autoimmune cell migration and invasion into the CNS and EAE development using the transfer EAE model. The major findings are that HIIT: 1. Does not alter the development of EAE in recipient mice via a direct neuroprotection; 2. Positively affects BBB integrity; 3. Results in increased migration of LNCs into the CNS as well as enhances demyelination of spinal cord sections; and 4. Attenuates the development of EAE via an immunomodulatory mechanism, which is associated with reductions in VLA-4 integrin expression and in the migratory capacity of encephalitogenic LNCs.

To our knowledge, the direct effects of HIIT on the CNS in autoimmune neuroinflammation has not been examined. First, our findings indicate that HIIT prior to autoimmune cell transfer does not affect the clinical course of EAE. Nevertheless, HIIT induced a substantial decrease in BBB permeability to biocytin-TMR tracer, indicating reduction in BBB disruption. This finding prompted us to further examine the impact of HIIT on BBB properties. Brain endothelial cells are characterized by structured tight junctions that are formed primarily by endothelial-specific proteins such as occludin and claudin-4^18,19^. Additionally, the expression of adhesion molecules at the BBB level is a pathogenic symptom in MS and EAE^20,21^. Inflamed CNS endothelial cells upregulate expression of both VCAM-1 and ICAM-1. VLA-4-VCAM-1 and LFA-1-ICAM-1 interactions are critically involved in the firm arrest and adherence of CD4+ T cells to the inflamed endothelium of the CNS vessels^22–31^. We previously showed that HICT interferes with VLA-4/VCAM-1 and LFA-1/ICAM-1 interactions^7^. However, while HIIT markedly inhibited the induction of VCAM-1 expression following encephalitogenic LNC transfer, it did not affect the expression of ICAM-1 adhesion molecule, nor the occludin and claudin-4 molecules, on CD31+ endothelial cells in the CNS of EAE mice. Hence, our findings suggest a partial effect of HIIT on the properties of the BBB. Interestingly, the number of PKH+ encephalitogenic LNCs in the CNS of HIIT recipient mice was higher than in sedentary controls. Taken together, our results indicate that HIIT does not induce a direct neuroprotective effect in EAE. Accordingly, a recent study showed that 12 weeks of HIIT did not influence the lipoprotein profile in MS brains^32^, supporting the notion that HIIT is less potent in directly affecting CNS pathological processes.

The finding that the HIIT-mediated improvement in BBB integrity was associated with increased migration of LNCs into the CNS and enhanced demyelination of spinal cord sections was surprising. Even more surprising was that the latter findings were not associated with an even more negative clinical progression versus SED (see Fig. 1). When comparing the effects of HIIT and HICT on the CNS one should consider that the metabolic effects are likely to differ markedly between the two paradigms. The higher exercise intensity in HIIT is likely to coincide with a greater production of, e.g., reactive oxygen species (ROS)^33^, increased lactate levels (decreases in blood pH)^34^, as well as higher catecholamine levels^35^. The extent to which excessive levels of such factors (or other exerkines) could affect on the CNS is not clear.

In this context, the ROS component is of interest, as it has been shown to contribute to microglial neurotoxicity, demyelination, and neuronal death^36–40^. Accordingly, there is evidence that mitochondria in neurons produce ROS, and that mitochondrial ROS overproduction and changes in mitochondrial redox homeostasis have been shown to be involved in a number of neurodegenerative diseases, such as Alzheimer’s disease, Parkinson’s disease and Amyotrophic Lateral Sclerosis^41^. Previously we showed that HICT had a large positive effect on the development of EAE both via direct neuroprotection and systemic immunomodulation^6,8^. These positive effects were associated with a decreased production of ROS by isolated neuroglia following exposure to PLP^8^. Similarly, it was demonstrated that administration of a mitochondria-targeted antioxidant (Mito-Q) to EAE-SED mice diminished the clinical progression of the disease^42^. Thus, the negative effects of HIIT on the CNS and lack of positive effect on disease progression may be related to excessive ROS production. Therefore, the links between ROS, HIIT and CNS inflammation warrant future investigation.

The next question is why HIIT did not result in even a greater deterioration in terms of disease progression versus SED. To address this question, we examined the effect of HIIT on systemic immunomodulation. We observed a number of changes that could counter the negative effects observed in the CNS and thereby mitigate the clinical deterioration. The observation that HIIT positively affected the systemic immune system in donor mice to attenuate disease progression in recipient mice is consistent with this explanation.

Specifically, we found that transfer of PLP- reactive encephalitogenic LNCs obtained from HIIT mice resulted in reduction of PKH26+ autoimmune cell infiltration into the CNS recipient mice. Blocking the integrin-adhesion molecule interactions is an effective strategy to prevent CNS inflammation in MS and EAE^22,43^. Accordingly, we previously showed that the HICT paradigm inhibits several key molecules involved in encephalitogenic LNC migration^7^. We therefore hypothesized that HIIT may also interfere with induction of integrins on LNCs in the peripheral lymphoid system upon immunization with the PLP peptide. However, while HICT induced a reduction in both VLA-4 and LFA-1 expression on PLP-reactive LNCs in vitro^7^, the HIIT paradigm induced an inhibition only on the VLA-4 integrin expression in LNCs and T cells*.*

Extensive research has been conducted on the role of VLA-4 integrin in leukocyte recruitment to the inflamed CNS during EAE^44^. The VLA-4 integrin was shown to mediate initial recruitment of encephalitogenic T cells, as well as the secondary infiltration of inflammatory cells, thereby contributing to and sustaining CNS inflammation^45^. Thus, our findings suggest that the inhibitory effect of HIIT on the expression of VLA-4 in PLP-reactive encephalitogenic LNCs contributes to the decrease in inflammatory infiltration into the CNS and attenuation of the clinical severity of EAE. Interestingly, VLA-4 expression in F4/80+ macrophages was not affected by HIIT, suggesting that the inhibitory effect of HIIT may be targeted to the T cell populations.

Although the number of injected encephalitogenic LNCs from donors to recipient groups was equal, we cannot rule out that the reduction in cell infiltration in the CNS occurred due to differences in post-injection proliferation between groups. Similarly, we cannot exclude other post-injection phenomena, such as differences in apoptosis to account for differences in T cell infiltration between groups. Other possibilities worth investigating in the future are changes in the differentiation and profile of the transferred cells in the CNS between the groups.

Of particular interest in this respect is the observation that although HIIT in donor mice impacts positively on disease progression in recipient mice, the effect is not as large as seen with HICT^6^. Again, this may be related to excessive ROS production during HIIT. The partial effects of HIIT on integrin expression may also, partially, explain the milder inhibitory effects of HIIT on EAE clinical course compared to the HICT program. Moreover, we demonstrated that while HICT causes a marked inhibition of PLP-induced T-cell proliferation without affecting the T-cell profile, HIIT does not alter T-cell proliferation, but rather inhibits polarization of T cells into T-helper 1 and T-helper 17 autoreactive populations^6^. We concluded that HICT and HIIT attenuate systemic autoimmunity and T cell encephalitogenicity by distinct immunomodulatory mechanisms. Thus, the current work corroborates the beneficial systemic immunomodulatory effects of HIIT in attenuation of EAE and further highlights the variations between HICT and HIIT paradigms.

**Figure 5:**
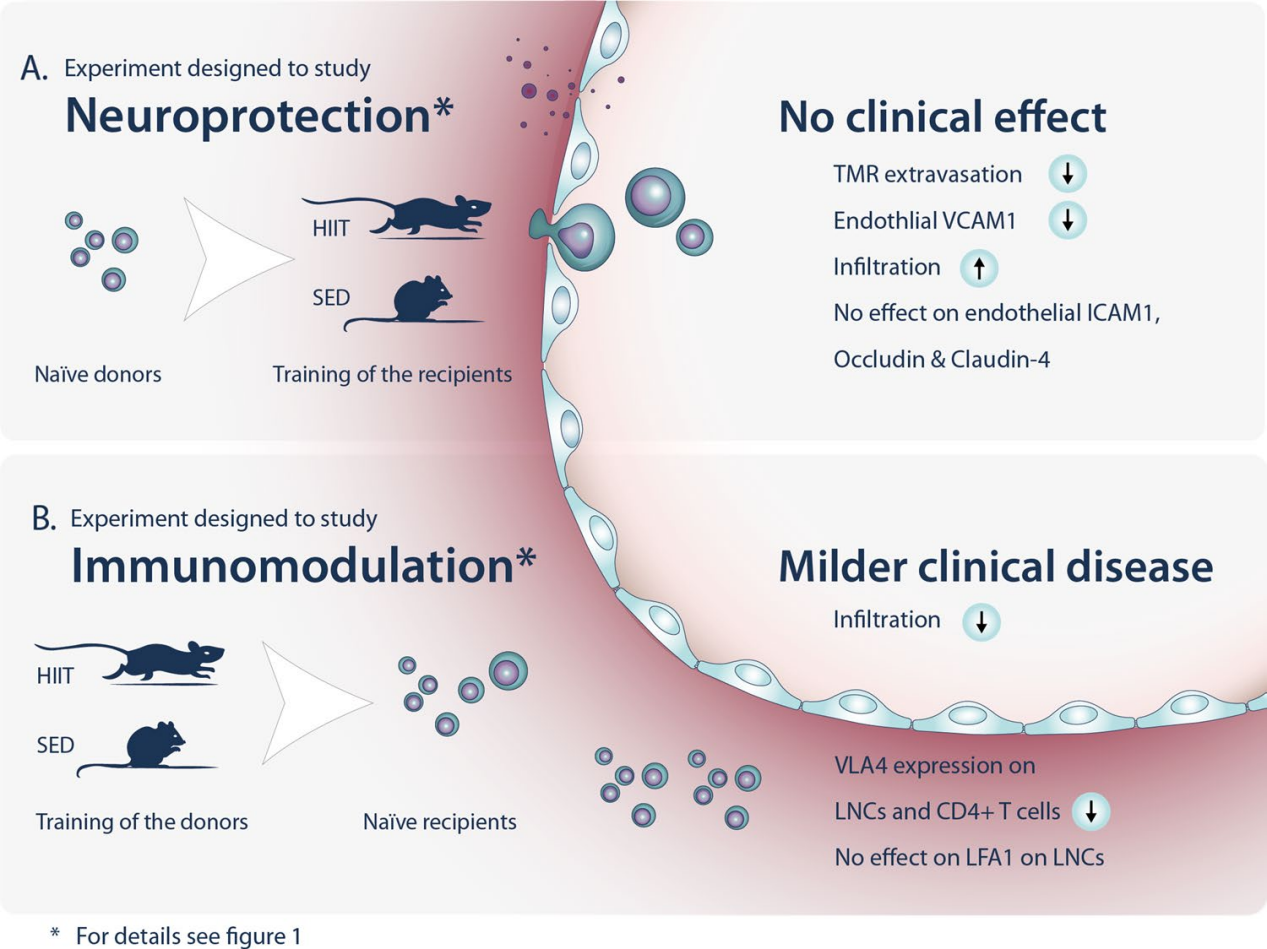
High-intensity interval training (HIIT) attenuates experimental autoimmune encephalomyelitis solely by immunomodulation. (**A**) To study neuroprotection, healthy recipient mice were subjected to a HIIT treadmill running program or sedentary (SED) period and injected with proteolipid protein (PLP)- reactive lymph node cells (LNCs) to develop EAE. HIIT did not affect the clinical course of EAE, compared to SED controls. EAE mice that underwent HIIT prior to EAE induction exhibited a reduction in 5-(and 6)-tetramethylrhodamine (TMR) extravasation and endothelial vascular cell adhesion molecule (VCAM)-1 expression, and an increased infiltration of autoimmune cells into their CNS. No effect was observed on endothelial intercellular adhesion molecule (ICAM)-1, occludin and claudin-4 expressions. (**B**) To study immunomodulation, healthy donor mice were subjected to a HIIT treadmill running program or SED period, followed by immunization with PLP peptide. HIIT or SED derived- PLP-reactive autoimmune cells were injected into naïve recipients that developed EAE. EAE mice that were injected with HIIT- derived autoimmune cells exhibited milder EAE clinical course and reduced autoimmune cell infiltration in their CNS. HIIT induced reductions in very late antigen (VLA)-4 expression on LNCs and CD4+ T cells but did not affect the expression of LFA-1 on LNCs. Thus, HIIT attenuates EAE only by an immunomodulatory effect.

In conclusion, HIIT does not induce direct neuroprotective effects in an experimental model of autoimmune neuroinflammation but induces systemic immunomodulation to attenuate EAE (Fig. 5). Our results indicate that HIIT affects the migration and invasion of encephalitogenic LNCs by affecting the systemic immune system with an associated reduction of VLA-4 expression in PLP-reactive encephalitogenic cells. While we previously found that the effects of HICT on EAE are mediated by ~ 70% by immunomodulation and ~ 30% by direct neuroprotection^7^, the current study demonstrates that the beneficial effect of HIIT stems solely from systemic immunomodulation. Thus, the clinical implication of the findings in the present study is that HICT is superior to HIIT in terms of impacting positively on disease progression, possibly because of differences in ROS production or other metabolic factors. This highlights the need for a systematic comparisons of various training paradigms for designing effective clinical treatments in MS patients and other patients with autoimmune diseases.

## Data Availability

No supporting data besides presented in the manuscript.

## References

[1] Heine M (2016). Cardiopulmonary fitness is related to disease severity in multiple sclerosis. Mult. Scler..

[2] Motl RW (2020). Exercise and multiple sclerosis. Adv. Exp. Med. Biol..

[3] Einstein O, Katz A, Ben-Hur T (2022). Physical exercise therapy for autoimmune neuroinflammation: Application of knowledge from animal models to patient care. Autoimmun. Rev..

[4] Einstein O (2018). Exercise training attenuates experimental autoimmune encephalomyelitis by peripheral immunomodulation rather than direct neuroprotection. Exp. Neurol..

[5] Fainstein N (2019). Exercise intensity-dependent immunomodulatory effects on encephalomyelitis. Ann. Clin. Transl. Neurol..

[6] Goldberg Y (2021). Continuous and interval training attenuate encephalomyelitis by separate immunomodulatory mechanisms. Ann. Clin. Transl. Neurol..

[7] Hamdi L (2022). Exercise training alters autoimmune cell invasion into the brain in autoimmune encephalomyelitis. Ann. Clin. Transl. Neurol..

[8] Zaychik Y (2021). High-intensity exercise training protects the brain against autoimmune neuroinflammation: Regulation of microglial redox and pro-inflammatory functions. Front. Cell. Neurosci..

[9] Jacob N (2023). Effects of high-intensity interval training protocols on blood lactate levels and cognition in healthy adults: Systematic review and meta-regression. Sports Med..

[10] Elliott AD, Rajopadhyaya K, Bentley DJ, Beltrame JF, Aromataris EC (2015). Interval training versus continuous exercise in patients with coronary artery disease: A meta-analysis. Heart Lung Circ..

[11] Milanovic Z, Sporis G, Weston M (2015). Effectiveness of high-intensity interval training (HIT) and continuous endurance training for VO2max improvements: A systematic review and meta-analysis of controlled trials. Sports Med..

[12] Weston KS, Wisloff U, Coombes JS (2014). High-intensity interval training in patients with lifestyle-induced cardiometabolic disease: A systematic review and meta-analysis. Br. J. Sports Med..

[13] Bartlett DB (2018). Ten weeks of high-intensity interval walk training is associated with reduced disease activity and improved innate immune function in older adults with rheumatoid arthritis: A pilot study. Arthritis Res. Ther..

[14] De Nardi AT, Tolves T, Lenzi TL, Signori LU, Silva A (2018). High-intensity interval training versus continuous training on physiological and metabolic variables in prediabetes and type 2 diabetes: A meta-analysis. Diabetes Res. Clin. Pract..

[15] Stoa EM (2017). High-intensity aerobic interval training improves aerobic fitness and HbA1c among persons diagnosed with type 2 diabetes. Eur. J. Appl. Physiol..

[16] Campbell E, Coulter EH, Paul L (2018). High intensity interval training for people with multiple sclerosis: A systematic review. Mult. Scler. Relat. Disord..

[17] Theotokis P (2016). Nogo receptor complex expression dynamics in the inflammatory foci of central nervous system experimental autoimmune demyelination. J. Neuroinflammation.

[18] Gunzel D, Fromm M (2012). Claudins and other tight junction proteins. Compr. Physiol..

[19] Heinemann U, Schuetz A (2019). Structural features of tight-junction proteins. Int. J. Mol. Sci..

[20] Cannella B, Raine CS (1995). The adhesion molecule and cytokine profile of multiple sclerosis lesions. Ann. Neurol..

[21] Engelhardt B, Conley FK, Butcher EC (1994). Cell adhesion molecules on vessels during inflammation in the mouse central nervous system. J. Neuroimmunol..

[22] Archelos JJ (1993). Inhibition of experimental autoimmune encephalomyelitis by an antibody to the intercellular adhesion molecule ICAM-1. Ann. Neurol..

[23] Bullard DC (2007). Intercellular adhesion molecule-1 expression is required on multiple cell types for the development of experimental autoimmune encephalomyelitis. J. Immunol..

[24] Carman CV, Martinelli R (2015). T lymphocyte-endothelial interactions: Emerging understanding of trafficking and antigen-specific immunity. Front. Immunol..

[25] Gahmberg CG (2009). Regulation of integrin activity and signalling. Biochim. Biophys. Acta.

[26] Kerfoot SM, Kubes P (2002). Overlapping roles of P-selectin and alpha 4 integrin to recruit leukocytes to the central nervous system in experimental autoimmune encephalomyelitis. J. Immunol..

[27] Laschinger M, Vajkoczy P, Engelhardt B (2002). Encephalitogenic T cells use LFA-1 for transendothelial migration but not during capture and initial adhesion strengthening in healthy spinal cord microvessels in vivo. Eur. J. Immunol..

[28] Morrissey SP (1996). Partial inhibition of AT-EAE by an antibody to ICAM-1: Clinico-histological and MRI studies. J. Neuroimmunol..

[29] Rothhammer V (2011). Th17 lymphocytes traffic to the central nervous system independently of alpha4 integrin expression during EAE. J. Exp. Med..

[30] Vajkoczy P, Laschinger M, Engelhardt B (2001). Alpha4-integrin-VCAM-1 binding mediates G protein-independent capture of encephalitogenic T cell blasts to CNS white matter microvessels. J. Clin. Invest..

[31] Xie C (2006). Suppression of experimental autoimmune encephalomyelitis by extracellular adherence protein of Staphylococcus aureus. J. Exp. Med..

[32] Jorissen W (2018). Twelve weeks of medium-intensity exercise therapy affects the lipoprotein profile of multiple sclerosis patients. Int. J. Mol. Sci..

[33] Zhang SJ (2007). Activation of aconitase in mouse fast-twitch skeletal muscle during contraction-mediated oxidative stress. Am. J. Physiol. Cell Physiol..

[34] Sahlin K, Katz A, Henriksson J (1987). Redox state and lactate accumulation in human skeletal muscle during dynamic exercise. Biochem. J..

[35] Nalbandian HM, Radak Z, Takeda M (2018). Effects of active recovery during interval training on plasma catecholamines and insulin. J. Sports Med. Phys. Fit..

[36] Gilgun-Sherki Y, Melamed E, Offen D (2004). The role of oxidative stress in the pathogenesis of multiple sclerosis: The need for effective antioxidant therapy. J. Neurol..

[37] Heneka MT, Kummer MP, Latz E (2014). Innate immune activation in neurodegenerative disease. Nat. Rev. Immunol..

[38] Martindale JL, Holbrook NJ (2002). Cellular response to oxidative stress: Signaling for suicide and survival. J. Cell. Physiol..

[39] Uttara B, Singh AV, Zamboni P, Mahajan RT (2009). Oxidative stress and neurodegenerative diseases: A review of upstream and downstream antioxidant therapeutic options. Curr. Neuropharmacol..

[40] van der Goes A (1998). Reactive oxygen species are required for the phagocytosis of myelin by macrophages. J. Neuroimmunol..

[41] Angelova PR, Abramov AY (2018). Role of mitochondrial ROS in the brain: From physiology to neurodegeneration. FEBS Lett..

[42] Mao P, Manczak M, Shirendeb UP, Reddy PH (1832). MitoQ, a mitochondria-targeted antioxidant, delays disease progression and alleviates pathogenesis in an experimental autoimmune encephalomyelitis mouse model of multiple sclerosis. Biochim. Biophys. Acta.

[43] Steinman L (2005). Blocking adhesion molecules as therapy for multiple sclerosis: Natalizumab. Nat. Rev. Drug Discov..

[44] Sheremata WA, Minagar A, Alexander JS, Vollmer T (2005). The role of alpha-4 integrin in the aetiology of multiple sclerosis: Current knowledge and therapeutic implications. CNS Drugs.

[45] Brocke S, Piercy C, Steinman L, Weissman IL, Veromaa T (1999). Antibodies to CD44 and integrin alpha4, but not L-selectin, prevent central nervous system inflammation and experimental encephalomyelitis by blocking secondary leukocyte recruitment. Proc. Natl. Acad. Sci. U.S.A..

